# Major James Moir

**Published:** 1913-11-01

**Authors:** 


### Obituary.

Major James Moir, R.A.M.C., has died at Felixstowe, at the age of fifty-three. Graduating M.B., C.M. at Aberdeen University in 1883, he entered the Royal Army Medical Corps three years later, and saw active service in the South African War. For this he received the Queen's Medal, with three clasps. He retired in 1906.

Deputy Surgeon-General Nathaniel Norris, formerly of the Indian Medical Service, has died at Chester aged eighty-two. Qualifying L.S.A. and M.R.C.S. in 1854, he entered the Army in the following year, and saw active service with the Jowaki Expedition in 1877, in the Afghan War of 1878-80, and was, we believe, the first medical inspector on the lines of communication in the Nile Expedition four years later.

